# Low Dietary Protein Intakes and Associated Dietary Patterns and Functional Limitations in an Aging Population: A NHANES Analysis

**DOI:** 10.1007/s12603-019-1174-1

**Published:** 2019-02-19

**Authors:** J.L. Krok-Schoen, A. Archdeacon Price, M. Luo, O.J. Kelly, Christopher Alan Taylor

**Affiliations:** 1 Ohio State University, Columbus, Ohio, USA; 2 Abbott Nutrition, Columbus, Ohio, USA

**Keywords:** Diet quality, protein intakes, physical limitations

## Abstract

**Objective:**

To investigate protein intakes across demographic characteristics in relation to dietary patterns and functional outcomes in older adults.

**Design:**

Observational and cross-sectional study.

**Setting:**

Non-institutionalized participants from the 2005–2014 National Health and Nutrition Examination Survey.

**Participants:**

Data from 11,680 adults were categorized into 51–60 years (n= 4,016), 61–70 years (n=3,854), and 71 years and older (n=3,810) for analysis.

**Measurements:**

Adults were stratified by meeting or not meeting the protein recommendation (0.8 g/kg/d) to compare demographics, diet quality with Healthy Eating Index– 2015, functional limitations, and other dietary intakes. Dietary recalls were collected using the multiple pass method. Data analyses were weighted to create a nationally–representative sample.

**Results:**

Dietary protein intakes were significantly lower in older age groups, with up to 46% of the oldest adults not meeting the protein intake recommendation. Participants consuming protein below the recommended intake level had significantly poorer diet quality across all age groups (P<0.01), however, overall diet quality was better in older adults. Those not meeting the protein recommendation were more likely to have intakes of other nutrients below recommended levels. Those below the protein recommendation had significantly more functional limitations across all age groups, while grip strength was significantly lower in those over 70 years old.

**Conclusion:**

Lower protein intakes, and lower diet quality and physical functioning are related in an aging population. Meeting the protein recommendation was linked to better overall diet quality and may be protective of lean mass; therefore, evaluation of individual characteristics which may affect protein intakes is crucial in supporting older adults to meet their protein needs.

## Introduction

Diet and nutrition play important roles in supporting healthful aging. Optimal nutrient intakes, especially dietary protein, are critically important for older adults as aging is associated with sarcopenia, a gradual and progressive decline in muscle mass, strength, and endurance (1). Sarcopenia can develop as early as 40 years of age and progresses considerably over the remainder of one’s life, resulting in a 50% or greater loss of muscle strength. In older adults, sarcopenia contributes to increased risks of falls and fracture and lower quality of life (2). Therefore, meeting protein as well as other nutrient needs is imperative in maintaining lean body mass and for preserving strength and functional abilities in aging (3, 4).

Prior assessments of age–related differences in protein intakes show that protein consumption was greatest in adults aged 19–30 years (mean 91 g per day), but lowest for older adults with mean protein intakes of 66 g per day (5). In general, older adults are consuming less food, which contributes to insufficient amounts of dietary protein and energy (6, 7). Several factors influence protein intakes in older adults, including reduced energy needs, genetic predispositions to low appetite, age– and disease–related anorexia, physical and mental disabilities that limit acquiring and preparing food, change in food preference, dysphagia, dental issues, and food insecurity due to financial and social limitations (6, 8). However, there remains a paucity of data regarding the proportion of older adults meeting recommended protein intake levels how protein intakes relate to overall diet quality and physical functioning. This is increasingly relevant as quality of life in aging is an important consideration.

While many are not meeting the current protein recommendation, several factors contribute to greater protein needs in older adults, suggesting those with lower protein intakes are even further away from optimal intakes. Body composition and lifestyle changes and comorbidities (9, 10, 11, 12), as well as insulin resistance which may drive a decreased sensitivity to dietary amino acids (13), and may all result in older people requiring more than the recommended protein level. From a physiological perspective, older adults may develop anabolic resistance, a reduced muscle protein synthesis rate response to protein intake (14). Furthermore, prolonged disuse of muscles and increased sedentary behavior could contribute to muscle atrophy among older adults (8, 14). Apart from low grade chronic inflammation associated with aging (15), chronic illness further elevates inflammation, especially for those with chronic obstructive pulmonary disease (COPD), chronic kidney disease, or heart failure, all of which require greater protein intakes (8). If dietary intakes of protein remain below the recommended intake with greater protein needs during aging, over time these could manifest as physical functional limitations. Understanding age–related differences in intakes could be pivotal for targeted nutritional interventions to promote optimal health outcomes in aging. Therefore, the purpose of this study was to examine protein intakes and associated dietary patterns relative to the protein intake recommendations and assess differences in functional limitations among a nationally representative sample of older aged adults.

## Methods

### Sample Population

Data from 11,680 adults over 51 years old from the 2005– 2014 National Health and Nutrition Examination Survey (NHANES) were utilized to examine protein intakes and associated dietary factors. NHANES collected data from the non–institutionalized population using a multistage stratified sampling technique to select participants. Demographic characteristics including age, gender, race, ethnicity, marital status, education level, and household income were obtained during in–home interviews. Physical and dietary measures were collected during visits to the mobile examination center. The National Center for Health Statistics’ Research Ethics Review Board reviewed and approved all data collection protocols.

### Dietary Quality and Patterns Measures

Dietary intakes were collected using the automated multiple pass method, which aims to collect foods and beverages consumed from midnight to midnight in the previous day by trained interviewers. Nutrient and MyPlate equivalent intakes were estimated by the US Department of Agriculture’s Food Surveys Research Group using the Food and Nutrients Database for Dietary Studies (16) and Food Pyramid Equivalents Database (17), respectively. To account for age–related differences in energy intakes, energy–adjusted nutrients were computed and presented as nutrient intakes per 1,000 kcal. Nutrient intakes were classified as above or below the estimated average requirement (EAR) or adequate intake (AI) from the day of intake (18). Meal skipping was categorized based the participants reporting of foods or beverages based from selfidentification of the meal occasion (breakfast, lunch, dinner).

Total protein intakes and measured body weight were used to classify participants as being above or below the individual dietary protein intake recommendation of 0.8 g/ kg/d. Differences in protein intakes from the recommendations were computed as the difference of intakes from recommended intakes.

To assess overall diet quality, the Healthy Eating Index (HEI) 2015 (19) was computed to assess intakes related to the 2015–2020 Dietary Guidelines for Americans (20). Higher scores on each of the 13 subscales are indicative of better diet quality, with the total scale ranging from 0–100, with higher scores denoting better diet quality.

### Physical Functioning

Physical functioning was assessed (yes/no) with the 19–item physical functioning questionnaire during an in–home interview to assess the participant’s level of disability. Physical functioning items included fine motor limitations (using a fork, knife, drinking from a cup, grasping small objects), gross motor limitations (preparing meals, walking and standing for long periods), and social limitations (going out to movies, attending a social event). Isometric grip strength (kg) was assessed with a hand grip dynanamometer. Grip strength was determined by summing the average values of grip strength for both hands.

### Analyses

Data from adults aged 51 years and older were categorized into 51–60 years, 61–70 years, and 71 years and older for analysis. Adults were then stratified by either meeting or not the recommended 0.8 g of protein/kg of body weight/d (0.8 g/kg/d) of protein to compare demographic characteristics, overall diet quality, nutrient intakes, physical functioning, and grip strength.

NHANES data were imported into SPSS Complex Samples (version 24, IBM SPPS Inc., Chicago, IL) for statistical analyses and to produce nationally representative estimates with statistically appropriate standard errors. Data are presented as means and standard errors or unweighted counts and weighted population percent for categorical data. Analysis of covariance for protein intakes and HEI scores were controlled for race/ ethnicity, gender, marital status, and percent of the federal poverty rate.

## Results

### Demographics

Complete dietary intake and anthropometric data from adults aged 51 years and older were categorized into 51–60 years (n=4,016), 61–70 years (n=3,854), and 71 years and older (n=3,810) were included in this analysis (Table 1). Across all age categories, males (69% of 51–60 year old, 63% of 61–70 year old, and 58% of those over 70 years old) are more likely to meet recommended protein intakes compared to females (55% of 51–60 year old’s, 52% of 61–70 year old, and 50% of those over 70 years old). Non–Hispanic blacks and those who were single, divorced, or widowed were least likely to meet recommended protein intakes across all age categories.

**Table 1:** Personal and demographic characteristics in US older adults, stratified by intakes above or below protein intake recommendations from the dietary recalla

		**51–60 yrs**		**61–70 yrs**		**>70 yrs**	
**Characteristic**	**Category**	**Below protein recommendation**	**Met protein recommendation**	**Below protein recommendation**	**Met protein recommendation**	**Below protein recommendation**	**Met protein recommendation**
				**n (%)**			
Gender	Male	646 (30.6%)	l340 (69.4%)	770 (36.6%)	1145 (63.4%)	842 (41.7%)	1051 (58.3%)
	Female	998 (45%)	l032 (55%)	1011 (48.4%)	928 (51.6%)	999 (50.3%)	918 (49.7%)
Race/Ethnicity	Mexican American	223 (36.4%)	374 (63.6%)	269 (42.8%)	356 (57.2%)	135 (49.7%)	133 (50.3%)
	Other Hispanic	l52 (39.4%)	237 (60.6%)	182 (42.5%)	211 (57.5%)	101 (45.8%)	97 (54.2%)
	Non–Hispanic White	657 (36.5%)	l037 (63.5%)	696 (41.4%)	922 (58.6%)	1202 (45.7%)	1385 (54.3%)
	Non–Hispanic Black	5l8 (52.9%)	485 (47.1%)	553 (57.9%)	426 (42.1%)	336 (57.4%)	247 (42.6%)
	Other or Multiracial	94 (27.6%)	239 (72.4%)	81 (38.1%)	158 (61.9%)	67 (42.5%)	107 (57.5%)
Marital Status	Single/Divorced/Widowed	683 (40.8%)	830 (59.2%)	744 (47.4%)	728 (52.6%)	862 (45.8%)	919 (54.2%)
	Married/Living as Married	96l (36.6%)	l542 (63.4%)	1037 (40.8%)	1345 (59.2%)	979 (47.4%)	1050 (52.6%)
Highest Education Completion	<9th grade	l88 (37.5%)	275 (62.5%)	293 (49.4%)	304 (50.6%)	380 (56%)	313 (44%)
	9–llth grade	256 (43.5%)	3l6 (56.5%)	310 (50.9%)	283 (49.1%)	320 (50.3%)	292 (49.7%)
	HS/GED	398 (42.6%)	532 (57.4%)	428 (46.3%)	457 (53.7%)	468 (48.2%)	489 (51.8%)
	Some college or Associate’s degree	468 (40.9%)	643 (59.1%)	467 (47%)	542 (53%)	400 (46.5%)	452 (53.5%)
	College grad	333 (29.7%)	605 (70.3%)	283 (31.8%)	485 (68.2%)	268 (36.4%)	418 (63.6%)
Meal consumption (% consuming)	Breakfast	l386 (86.8%)	2137 (91.5%)	1555 (87.9%)	1917 (94.2%)	1705 (94.4%)	1904 (97.1%)
	Lunch	lll7 (7l.7%)	1861 (84.3%)	1182 (73.4%)	1583 (81.9%)	1250 (71.6%)	1526 (81.3%)
	Dinner	l426 (90%)	2175 (94.9%)	1525 (90.9%)	1909 (96%)	1614 (90.4%)	1857 (95.9%)
	Snack	l563 (96.9%)	2275 (97.3%)	1672 (96.1%)	1969 (96.5%)	1697 (92.9%)	1848 (94.1%)
Number of meals reported	l meal	l65 (7.6%)	99 (3%)	155 (6.3%)	75 (2.3%)	111 (3.7%)	22 (0.6%)
	2 meals	646 (35%)	730 (22.8%)	726 (33.7%)	651 (23.2%)	714 (35.8%)	573 (24.4%)
	3 meals	824 (57%)	1538 (74%)	885 (59.5%)	1344 (74.5%)	1010 (60.4%)	1373 (74.9%)
				**Mean (SE)**			
Poverty–Income Ratio		3.0 (0.09)	3.6 (0.06)	3.0 (0.06)	3.4 (0.06)	2.5 (0.05)	2.8 (0.06)
Body Mass Index (kg/m^2^)		32.3 (0.27)	27.9 (0.18)	32 (0.22)	27.7 (0.18)	30.0 (0.19)	26.3 (0.12)
Weight (kg)		9l.3 (0.8)	80.4 (0.64)	89.4 (0.71)	78.5 (0.6)	80.5 (0.55)	71.3 (0.41)


a. Protein intake recommendations computed as 0.8 g of dietary protein per kilogram of body weight.

### Dietary Intakes

Across all age groups, those not meeting the recommended protein intake had significantly lower intakes of total energy (P<0.001) and energy adjusted protein (P<0.001, Table 2). Those not meeting the recommended protein intake requirement also had a significantly lower energy adjusted consumption of carbohydrates, total fat, saturated fat, monounsaturated fat, choline, vitamin C, phosphorus, zinc, and selenium per 1,000 kcal across all age groups. However, only 10% of adults 51–60 years old, 12% of adults 61–70 years old, and 13% of adults over 71 years old below the protein intake recommendation also had carbohydrates intakes below the AI (Table 3). Those not meeting the protein intake recommendation were also below the EAR for zinc, selenium, vitamin E, vitamin C, and vitamin D intakes across all age categories (Table 3). In addition, only 11% of adults 51–60 and 61–70 years old, and 13% of adults over 71 years old not meeting the protein recommendation were below the EAR for iron.

**Table 2:** Differences in energy and energy–adjusted nutrient intakes across age groups for those who were below or met the protein intake recommendation from the 2005–2014 National Health and Nutrition Examination Survey (NHANES) a,b,c

	**51–60 yr**			**61–70 yr**			**>70 yr**		
	**Below protein recommendation**	**Met protein recommendation**	**P**	**Below protein recommendation**	**Met protein recommendation**	**P**	**Below protein recommendation**	**Met protein recommendation**	**P**
Energy (kcal)	1630 (24)	2420 (23)	<0.001	1501 (17)	2213 (22)	<0.001	1356 (18)	1994 (22)	<0.001
Protein, total (g)	54.3 (0.8)	100.5 (1.1)	<0.001	51.0 (0.7)	93.0 (0.8)	<0.001	47.4 (0.6)	84.6 (0.8)	<0.001
Energy–adjusted intakes									
Protein (g)	35.0 (0.4)	43.4 (0.4)	<0.001	35.8 (0.4)	43.8 (0.4)	<0.001	36.5 (0.3)	44.2 (0.3)	<0.001
Carbohydrate (g)	128 (1.0)	115 (0.9)	<0.001	128 (1.0)	117 (0.8)	<0.001	133 (0.8)	122 (0.7)	<0.001
Dietary fiber (g)	8.6 (0.2)	8.4 (0.2)	0.564	9.4 (0.2)	9.3 (0.2)	0.361	9.8 (0.2)	9.7 (0.2)	0.342
Total fat (g)	36.2 (0.4)	38.8 (0.3)	<0.001	36.9 (0.3)	38.1 (0.3)	0.004	35.9 (0.3)	37.3 (0.3)	<0.001
Saturated fat (g)	11.3 (0.2)	12.4 (0.1)	<0.001	11.7 (0.2)	12.2 (0.1)	0.014	11.6 (0.1)	11.9 (0.2)	0.160
Monounsaturated fat (g)	12.9 (0.2)	14.0 (0.1)	<0.001	13.3 (0.2)	13.7 (0.1)	0.036	12.8 (0.1)	13.6 (0.1)	<0.001
Polyunsaturated fat (g)	8.7 (0.2)	8.9 (0.1)	0.246	8.7 (0.1)	8.7 (0.1)	0.672	8.3 (0.1)	8.6 (0.1)	0.158
Vitamin A, RAE (*μ*g)	311 (15.9)	339 (10.6)	0.076	358 (12.0)	381 (26.4)	0.413	405 (14.7)	402 (10.4)	0.854
Folate (*μ*g DFE)	268 (6.3)	257 (5.5)	0.239	273 (4.8)	268 (5.5)	0.602	308 (5.9)	286 (5.9)	0.005
Choline (mg)	153 (3.1)	171 (1.9)	<0.001	161 (3.0)	183 (2.5)	<0.001	165 (2.3)	184 (1.9)	<0.001
Vitamin B12 (*μ*g)	2.4 (0.1)	2.5 (0.1)	0.456	2.4 (0.1)	3.2 (0.3)	0.019	2.6 (0.1)	3.0 (0.1)	0.003
Vitamin C (mg)	49.4 (2.8)	43.0 (1.3)	0.040	51.6 (1.7)	45.2 (1.9)	0.012	57.5 (2.0)	50.9 (1.6)	0.005
Vitamin D (*μ*g)	2.1 (0.2)	2.4 (0.1)	0.083	2.2 (0.1)	2.6 (0.1)	0.004	2.6 (0.1)	3.2 (0.1)	<0.001
Vitamin K (*μ*g)	62.7 (3.7)	72.6 (4.7)	0.073	67.8 (3.0)	66.4 (3.1)	0.706	69.3 (3.9)	71.1 (3.3)	0.616
Calcium (mg)	432 (7.8)	458 (7.3)	0.023	466 (9.7)	468 (6.2)	0.819	479 (7.5)	497 (5.6)	0.031
Phosphorus (mg)	601 (6.9)	680 (5.7)	<0.001	636 (7.0)	702 (4.9)	<0.001	641 (5.0)	715 (5.3)	<0.001
Magnesium (mg)	150 (2.6)	153 (1.8)	0.315	158 (2.5)	160(2)	0.501	158 (2.2)	163 (2.0)	0.033
Iron (mg)	7.3 (0.2)	7.4 (0.1)	0.928	7.7 (0.1)	7.7 (0.1)	0.930	8.6 (0.1)	8.4 (0.1)	0.163
Zinc (mg)	4.9 (0.1)	5.9 (0.1)	<0.001	5.1 (0.1)	6.1 (0.1)	<0.001	5.6 (0.1)	6.4 (0.1)	<0.001
Copper (mg)	0.7 (0.02)	0.7 (0.01)	0.586	0.7 (0.01)	0.8 (0.05)	0.230	0.7 (0.02)	0.7 (0.01)	0.971
Sodium (mg)	1626 (20)	1731(23)	<0.001	1712 (21)	1727 (20)	0.597	1704 (18)	1718 (17)	0.487
Potassium (mg)	390 (36)	1372 (16)	0.655	1469 (20)	1442 (15)	0.341	1493 (18)	1515 (17)	0.273
Selenium (*μ*g)	49.4 (0.7)	58.7 (0.7)	<0.001	51.0 (0.7)	60.4 (0.7)	<0.001	51.2 (0.7)	59.7 (0.7)	<0.001


a. Mean (SE) intakes adjusted for race/ethnicity, gender, marital status and percent of federal poverty rate; b. Nutrients presented as energy–adjusted intakes per 1,000 kcal; c. Protein intake recommendations computed as 0.8 g of dietary protein per kilogram of body weight.

**Table 3:** Proportion below the Estimated Average Requirement or Adequate Intakes across Age Categories and Meeting the Protein Recommendationa

	**51–60 yrs**		**61–70 yrs**		**>70 yrs**	
	**Below Protein recommedation**	**Met Protein recommedation**	**Below Protein recommedation**	**Met Protein recommedation**	**Below Protein recomendation**	**Met Protein recommedation**
Carbohydrate	185 (10.2%)	64 (2.5%)	236 (11.9%)	49 (2.4%)	251 (12.9%)	45 (1.9%)
Fiber	1504 (90.9%)	1814 (76.0%)	1665 (92.5%)	1586 (76.7%)	1738 (94.1%)	1603 (79.8%)
Thiamin	663 (35.1%)	246 (9.1%)	752 (36.2%)	213 (9.1%)	758 (39.0%)	198 (9.1%)
Riboflavin	414 (17.8%)	90 (2.6%)	481 (19.7%)	97 (2.7%)	447 (20%)	66 (2.7%)
Niacin	432 (21.3%)	45 (1.0%)	538 (25.5%)	50 (2.9%)	607 (30.5%)	64 (3.3%)
Vitamin B6	984 (54.9%)	365 (14.2%)	1097 (57.7%)	352 (15.9%)	1116 (57.5%)	396 (19.8%)
Folate	772 (41.9%)	432 (17.2%)	887 (45.8%)	388 (17.7%)	906 (47.2%)	422 (20.7%)
Vitamin B12	702 (38.6%)	291 (11.2%)	721 (36.4%)	271 (11.3%)	699 (35.3%)	185 (8.3%)
Vitamin C	1062 (63.6%)	1212 (49.5%)	1117 (62.3%)	1010 (48.0%)	1092 (57.6%)	913 (45.0%)
Choline	1590 (96.2%)	1762 (76.2%)	1739 (97.8%)	1599 (77.5%)	1819 (98.6%)	1666 (84.2%)
Vitamin A	1206 (68.9%)	1171 (42.0%)	1251 (66.3%)	994 (42.6%)	1246 (64.3%)	807 (37.7%)
Vitamin D	1370 (96.4%)	1710 (84.5%)	1463 (97.0%)	1481 (85.5%)	1475 (96.7%)	1369 (84.8%)
Vitamin E	1541 (93.0%)	1835 (74.0%)	1670 (92.1%)	1659 (77.5%)	1754 (94.0%)	1650 (83.5%)
Vitamin K	1288 (75.5%)	1526 (59.8%)	1422 (76.4%)	1325 (60.4%)	1512 (80.5%)	1325 (64.8%)
Calcium	1337 (80.5%)	1105 (43.5%)	1430 (77.1%)	1060 (47.1%)	1625 (87.7%)	1161 (57.5%)
Phosphorus	316 (15.0%)	7 (0.2%)	355 (17.1%)	8 (0.2%)	425 (21.9%)	13 (0.7%)
Magnesium	1364 (81.2%)	1046 (39.6%)	1532 (82.9%)	984 (43.5%)	1661 (88.3%)	1075 (51.9%)
Iron	214 (10.8%)	20 (0.4%)	258 (10.7%)	21 (0.6%)	278 (13.3%)	24 (1.4%)
Zinc	1045 (58.7%)	387 (13.4%)	1171 (60.2%)	383 (15.2%)	1291 (66.4%)	408 (17.4%)
Copper	543 (29.1%)	146 (4.4%)	626 (31.7%)	127 (5.1%)	686 (34.6%)	157 (7.4%)
Selenium	337 (19.1%)	12 (0.5%)	390 (19.9%)	11 (0.6%)	475 (24.7%)	23 (1.2%)


a. Number; n (% of total). For nutrients without an EAR, the Adequate Intake is used, italicized above; b. Protein intake recommendation computed as 0.8 g of dietary protein per kilogram of body weight

Skipping meal frequencies for breakfast, lunch and dinner were higher across each age group for adults who did not meet the protein intake recommendation. Approximately 74% of adults meeting the protein recommendation reported consuming three meals per day, compared to the 40–43% of those who did not meet the recommendation reporting fewer than three meals per day.

### Diet Quality

Across all age categories, adults not meeting the recommended protein intake had significantly lower total HEI scores (Table 4). Those not meeting protein recommendations had scored significantly less for greens and beans (P<0.001), dairy (P<0.001), total protein foods (P<0.001 for all age categories), seafood and plant protein foods (P<0.001), refined grains (P=0.022 for 51–60 year old’s, P=0.005 for 61–70 year old’s, P<0.001 for >70 year old’s) and added sugars (P<0.001 for all age categories), however, the whole grain score was only significantly lower for those not meeting the protein recommendation in the 61–70 year old’s, although whole grain intakes were low overall (highest mean score was 3.5/10 in the over 70 age group). Notably, adults aged 51–60 years old not meeting protein recommendations had significantly lower diet quality scores for sodium, which as a moderation subscale indicates they consumed more of this nutrient (P<0.001). Adults between 61–70 years old not meeting the protein recommendation had significantly lower diet quality scores for total fruit (P=0.007).

**Table 4:** Differences in Healthy Eating Index 2015 scores across age groups and in those below or meeting the protein intake recommendationa,b

	**51–60 yrs**			**61–70 yrs**			**>70 yrs**		
**HEI–2015 Score (score range)**	**Below protein recommendation**	**Met protein recommendation**	**P**	**Below protein recommendation**	**Met protein recommendation**	**P**	**Below protein recommendation**	**Met protein recommendation**	**P**
Total Fruit (0–5)	2.3 (0.11)	2.2 (0.06)	0.211	2.5 (0.08)	2.5 (0.07)	0.512	3.0 (0.07)	2.9 (0.07)	0.078
Whole Fruit (0–5)	2.3 (0.10)	2.3 (0.07)	0.691	2.4 (0.07)	2.7 (0.07)	0.007	2.8 (0.07)	2.9 (0.06)	0.260
Total Vegetables (0–5)	3.1 (0.08)	3.3 (0.05)	0.025	3.3 (0.06)	3.4 (0.05)	0.092	3.1 (0.06)	3.4 (0.05)	0.003
Greens & Beans (0–5)	1.3 (0.09)	1.8 (0.07)	<0.001	1.5 (0.09)	2.0 (0.08)	<0.001	1.3 (0.06)	1.8 (0.07)	<0.001
Whole Grains (0–10)	2.6 (0.14)	2.7 (0.13)	0.682	2.8 (0.13)	3.1 (0.12)	0.030	3.4 (0.13)	3.5 (0.11)	0.292
Dairy (0–10)	4.2 (0.15)	5.0 (0.11)	<0.001	4.4 (0.12)	5.2 (0.09)	<0.001	4.7 (0.1)	5.4 (0.09)	<0.001
Protein Foods (0–5)	4.0 (0.06)	4.6 (0.03)	<0.001	3.9 (0.05)	4.6 (0.04)	<0.001	3.9 (0.05)	4.6 (0.04)	<0.001
Seafood & Plant Proteins (0–5)	2.2 (0.08)	2.6 (0.07)	<0.001	2.4 (0.10)	2.9 (0.08)	<0.001	2.0 (0.06)	2.8 (0.08)	<0.001
Fatty Acids (0–10)	5.6 (0.15)	5.3 (0.08)	0.055	5.6 (0.13)	5.3 (0.12)	0.093	5.12 (0.12)	5.3 (0.13)	0.401
Refined Grains^c^ (0–10)	6.2 (0.15)	6.7 (0.14)	0.022	6.1 (0.12)	6.6 (0.14)	0.005	6.1 (0.11)	6.8 (0.10)	<0.001
Sodiumc (0–10)	4.9 (0.14)	4.2 (0.11)	<0.001	4.4 (0.14)	4.1 (0.12)	0.146	4.5 (0.12)	4.3 (0.11)	0.178
Added Sugars^c^ (0–10)	6.2 (0.19)	7.1 (0.10)	<0.001	6.7 (0.12)	7.6 (0.09)	<0.001	6.9 (0.11)	7.7 (0.09)	<0.001
Saturated Fat^c^ (0–10)	6.7 (0.14)	5.9 (0.11)	<0.001	6.4 (0.11)	6.1 (0.11)	0.036	6.5 (0.12)	6.3 (0.13)	0.177
Total HEI Score (0–100)	51.4 (0.68)	53.5 (0.41)	0.005	52.2 (0.53)	55.9 (0.53)	<0.001	53.3 (0.48)	57.6 (0.56)	<0.001


a. Mean (SE) intakes adjusted for race/ethnicity, gender, marital status and percent of federal poverty rate; b. Protein intake recommendation computed as 0.8 g of dietary protein per kilogram of body weight; c. Higher scores for scales related to moderation represent lower intakes.

### Functional Limitations

Across all age categories, those not meeting the protein recommendation more commonly encountered physical limitations than those meeting the protein recommendation. Specifically, those below the protein intake recommendation were more likely to be limited when stooping, crouching or kneeling, standing or sitting for long periods, walking up 10 steps, preparing meals, and walking for a quarter mile. The prevalence of physical limitations for adults aged 51–60 years old including: sitting for long periods, getting in and out of bed, preparing meals, leisure activities at home, and using a fork, knife, and drinking from a cup was within 3% of the prevalence of these same limitations for the sample population over 71 years old. Adults across every age category had a higher prevalence of physical, mental, and social limitations than those meeting the protein recommendation, except for adults over 71 years old. Combined hand grip strength was not significantly different for those not meeting the protein recommendation for participants aged 51–60 and 61–70 years, but was significantly lower for participants over 70 years.

## Discussion

The requirement for higher protein intake recommendations in the older population is being led by several experts in the field, both in the free living population (8, 11, 13) and in hospitalized patients (21). This study adds to the body of evidence supporting an emphasis on protein intakes in aging. Protein intakes and associated dietary patterns in relation to demographic, anthropometric and functional characteristics were examined, among older adults in the United States. A considerable proportion of older adults (31–50%) did not meet their protein recommendation (0.8 g/kg/d) on the day of their NHANES dietary assessment. This contradicts the common perception that Americans are usually meeting or exceeding the 0.8 g/kg/d protein recommendation. In the most recent study, Berryman and colleagues (22) suggest that protein is consumed in excess of the recommendation in the majority of people aged 2 years or older. These estimates were statistically adjusted for assumed intrapersonal variability and used 0.8 g/ kg/d of protein for ideal body weight to determine protein intake recommendations. These data suggest that 7–19% of older adults did not meet protein intake recommendations, nevertheless, this assessment of unadjusted protein intakes per ideal body weight suggests that the rate of low protein intakes on a given day is higher, nearly one third of older adults. The data reported here represent a single day’s intake, which cannot be assumed to be the usual intake pattern, however, if these nationally representative data reflect the broader pattern of protein intakes, it indicates further efforts to optimize dietary quality and protein intakes for aging are required.

Prevalence data within this analysis suggests that older age groups are progressively less likely to meet protein recommendations, the age range which corresponds with when sarcopenia becomes more prevalent (23), suggesting habitually moderate protein intakes may contribute to sarcopenia (11). If protein needs for older people exceed the current recommendation of 0.8 g/kg and over one third are not meeting that standard, these data suggest that far fewer older adults would be meeting the proposed higher levels (1–1.2 g/kg/d) needed to meet the demands to promote healthful aging (24). If the recommendations are increased, specific strategies will be needed to support older adults consuming diets, especially for those already below the existing 0.8 g/kg.

A major challenge to increasing nutrient intakes in aging is the appetite decline associated with age. This phenomenon has been attributed to numerous factors, such as poor dentition, reduced sense of taste and smell, delayed gastric emptying and depression, all of which are conditions prevalent among older adults (25, 26, 27, 28, 29). The anorexia of aging is well established, and is reported to affect between 15 and 30% of aging adults (30, 31). Apart from pharmacological appetite stimulants, addressing these issues may help to enhance appetite among older adults. Other approaches to increase nutrient intake in the older population include, improving oral health, enhance the sensory attributes of foods (flavor and smells) with extra seasonings, introducing variety of foods at mealtimes, utilizing colored cutlery and plating options, increasing physical activity, and enriching foods served with supplemental calories (27, 28, 32). The complexity of appetite in the older population may make increasing protein challenging, but it seems a combination of tactics may be required at the individual level.

Adults not meeting the protein recommendation were more likely to have lower intakes of several nutrients, including fiber, thiamin, niacin, vitamin B6, folate, choline, vitamin C, vitamin B12, vitamin A, vitamin D, vitamin E, vitamin K, zinc, calcium, phosphorus, magnesium, iron, copper, and selenium. The 2015–2020 Dietary Guidelines for Americans recognizes that within the context of a poorer quality diet (low in vegetables, fruits, whole grains, and dairy) certain nutrients are consumed below the EAR or AI level. These are referred to as nutrients of public health concern and include fiber, choline, vitamin C, vitamin A, vitamin D, vitamin E, potassium, calcium, magnesium and iron. Potassium is also a nutrient of public health concern but it did not feature in these analyses. Examining the function of each vitamin and mineral that was found to be lower in those not meeting the protein recommendation, in the context of healthy aging is beyond the scope of this paper, however, their physiological functions in human metabolism are well known; therefore, metabolic homeostasis may be affected with habitual insufficiencies of these nutrients with possible implications for aging outcomes. Of particular note dietary fiber is associated with many benefits, especially in reducing the risk for coronary heart disease (33); vitamin D inadequacy is associated with reduced mobility, and an increase in the risk for falls and fractures, and death from cardiovascular disease (34), and; zinc insufficiencies can result in lower zinc concentrations in immune cells and platelets and may cause dysfunctions in immunity, and reduce healing time (35), which could be troublesome for elderly populations. Overall, adults not meeting protein needs have much higher likelihood of lower micronutrient intakes (on the day of intake), and nutrient deficiencies, combined with a lower protein intake, in older adults may increase risks of common issues, such as falls, pressure sores, osteomalacia, osteoporosis, hip fractures, muscle weakness, and mortality (27, 31).

The current study found that diet quality (measured by HEI) was poorest in adults aged 51–60 years, compared to their older counterparts. This finding is in agreement with previous studies that found older adults had better quality diets than middle aged adults (24, 36, 37, 38); however, the mean HEI score in a sample of older adults (60 years and older) has been reported to be considerably higher (HEI: 66.6) than seen in this nationally representative study (39). However, these data were from earlier NHANES results (1999–2002) and used a slightly different coding system for the HEI score; although we expect no significant impact on the overall score using the revised coding. We hypothesize that age related differences related to habitual dietary patters, such as the frequencies of dining out and cooking meals, may contribute to the better diet quality in older adults compared to middle aged adults. Furthermore, older adults, may have consumed more wholesome diets, reflected in higher HEI scores, over their lifetime, which may have had long lasting effects, and those diets may have contributed to their longevity. The presence of low HEI scores, with increased prevalence of chronic diseases in the younger age group, suggests that a greater effort is required to improve the diet quality of the 51–60 year old’s to help reduce their risk of developing additional chronic conditions and improve their diet as they age. In light of the poor dietary habits of US adults in these data and in other analyses (40), and the rise of obesity and other chronic disease at even younger ages (41, 42, 43), lifelong dietary improvement strategies are needed to support healthful aging and disease prevention and treatment (30).

**Table 5:** Frequency of limitations and grip strength differences across age groups in those below or meeting the protein intake recommendationa,b

	**51–60 yrs**		**61–70 yrs**		**>70 yrs**	
**Limitations Experienced**	**Below protein recommendation**	**Met protein recommendation**	**Below protein recommendation**	**Met protein recommendation**	**Below protein recommendation**	**Met protein recommendation**
			*n (population %)*			
Stooping, crouching, kneeling	512 (27.8%)	427 (16.5%)	902 (50.2%)	754 (33.9%)	1054 (58.3%)	935 (46.8%)
Standing for long periods	458 (24.9%)	403 (16.1%)	732 (37.9%)	615 (25.6%)	950 (52.0%)	833 (41.4%)
Push or pull large objects	388 (21.1%)	353 (14.0%)	599 (32.1%)	501 (22.4%)	757 (41.2%)	666 (32.9%)
Sitting for long periods	336 (16.7%)	290 (11.2%)	436 (20.4%)	365 (14.5%)	292 (14.6%)	283 (13.1%)
House chore	310 (16.4%)	243 (9.3%)	418 (21.0%)	286 (11.1%)	459 (24.7%)	408 (19.6%)
Lifting or carrying	317 (15.7%)	244 (8.3%)	439 (21.1%)	363 (12.8%)	500 (26.7%)	463 (21.6%)
Standing up from armless chair	291 (15.3%)	210 (7.6%)	439 (21.3%)	325 (12.6%)	565 (29.9%)	471 (21.2%)
Going out to movies, events	264 (13.7%)	191 (7.0%)	331 (16.2%)	244 (9.0%)	399 (20.7%)	343 (15.8%)
Getting in and out of bed	269 (13.1%)	210 (6.6%)	322 (13.1%)	244 (8.0%)	324 (15.4%)	264 (11.6%)
Reaching up over head	255 (12.6%)	207 (6.7%)	337 (15.9%)	289 (10.8%)	389 (20.4%)	324 (15.5%)
Attending social event	206 (11.0%)	150 (5.8%)	255 (10.6%)	187 (7.0%)	287 (15.4%)	257 (11.2%)
Walking for a quarter mile	189 (11.0%)	175 (7.2%)	356 (23.6%)	264 (12.2%)	396 (29.5%)	327 (21.0%)
Grasp/holding small objects	201 (10.6%)	170 (6.5%)	281 (13.6%)	291 (12.5%)	338 (17.8%)	329 (16.4%)
Walking up ten steps	148 (9.3%)	131 (4.8%)	254 (17.4%)	178 (8.0%)	290 (21.4%)	220 (14.1%)
Dressing yourself	193 (9.3%)	147 (4.8%)	241 (9.8%)	192 (7.0%)	280 (14.2%)	241 (10.1%)
Preparing meals	154 (7.6%)	84 (2.8%)	156 (7.6%)	108 (4.1%)	185 (9.3%)	177 (8.0%)
Walking between rooms	147 (7.1%)	88 (2.7%)	183 (8.3%)	113 (3.7%)	263 (13.3%)	213 (9.5%)
Leisure activity at home	99 (4.8%)	79 (2.4%)	106 (4.5%)	96 (3.2%)	127 (6.0%)	103 (4.4%)
Using fork, knife, drinking from cup	73 (4.1%)	74 (2.3%)	106 (4.2%)	75 (2.8%)	129 (6.7%)	130 (5.6%)
			*Mean (SE)*			
Number of Limitations	2.2 (0.1)	1.6 (0.1)*	3.2 (0.1)	2.3 (0.1)*	4.2 (0.1)	3.5 (0.1)*
Combined Grip Strength (kg)	71.8 (1.1)	70.5 (0.7)	66.7 (0.8)	65.8 (0.6)	56.6 (0.6)	54.8 (0.6)*


a. Protein intake recommendation computed as 0.8 g of dietary protein per kilogram of body weight; b. Mean adjusted for race/ethnicity, gender, marital status and percent of federal poverty rate; ^Significantly (P<0.05) different between below or meeting the protein recommendation within the age category

This analysis found a positive association between achieving the recommended protein intake and self–reported physical functioning. The functional limitations associated with not meeting the protein requirement were all related to activities of daily living such as stooping, crouching or kneeling, standing or sitting for long periods, walking up 10 steps, preparing meals, and walking for a quarter mile. This agrees with findings from the Framingham Offspring Study where those with a protein intake less than the recommended level (0.8 g/kg/d) were associated with a higher risk of becoming dependent in one or more of the functional tasks assessed at follow up (heavy house work, walking half a mile, going up and down stairs, stooping/kneeling/crouching, and lifting heavy items) (44). Mishra et al., found no relationship between consuming >25 g protein at two or more meals and grip strength when protein intake was expressed in terms of ideal body weight and in an unadjusted model (45); the adjusted model only found a positive association for women. This suggests gender differences but women also had lower total daily intakes of protein although may be higher if adjusted for weight (not reported by the authors).

Higher protein diets have been shown to increase physical functioning, particularly with activities such as walking half a mile, going up and down stairs, stooping, kneeling, crouching and lifting heavy items (46). All which were more commonly experienced limitations for those not consuming adequate protein in this analysis. In addition, provision of protein beyond its recommendations is associated with fewer impairments in upper and lower extremity function than in those not meeting protein recommendations (47). Future research could explore the relationship between protein intakes and physical functioning longitudinally. However, habitual activity levels (exercise) will always be a confounder in these studies, as the combination of activity and higher protein is associated with a greater reduction in risk of functional loss (44).

There is a general consensus recommending dietary protein intakes of 1–1.2 g/kg/d for the healthy population and higher for older adults and individuals with acute or chronic disease (up to 2 g/kg/d) depending on the clinical condition, to maintain physical function (3, 8, 48, 49, 50, 51). Others suggest that older people should consume 0.4 g/kg/meal to limit muscle mass loss [4]. This is in line with other investigators who recommend more protein at each meal, and not focus on the total daily intake, as a means to maximize muscle protein synthesis throughout the day (52), with Mamerow et al. proposing that 30 g of high quality protein at breakfast, lunch and dinner will better stimulate muscle protein synthesis (53). However, if the protein recommendations are increased, the proportion of older adults below the recommendation will also increase. Therefore, strategies to help increase dietary protein could also be considered along with the recommendations. Given the heterogeneity of the older population in relation to activity level, body composition and chronic conditions, clinicians and dietitians’ may help promote higher protein intakes through personalized dietary recommendations accounting for individual needs, medical comorbidities, current medications, and current food intake (54, 55).

Another issue with increasing protein requirements is how to obtain it, especially at each meal. Active adults seem to use supplements with protein more often (56), indicating there may be a perception that more protein is only needed if one is engaged in regular activities. There are gaps in the literature regarding methods to increase dietary protein above 0.8 g/kg/d using food only; oral nutritional supplements (ONS), protein only or mixed, are the standard way to increase protein intakes in human studies. Therefore, when food alone is insufficient to meet a patient’s needs, ONS may provide a means to meet protein intake recommendations. ONS have already been shown to be beneficial for older adults by improving muscle mass and lower extremity functioning (57), and reestablish energy balance among older men and women (58, 59), as well as combatting weight loss (27) in nutritionally compromised individuals. To some extent, sarcopenia can be managed with protein supplementation (60), considering all the factors that are working to reduce food intake in aging. Overall, ONS may provide a plethora of benefits to the aging population and should be considered as a nutritional strategy for improving protein, and other nutrient, intakes.

While the results presented here suggest not meeting the recommended protein intake may be an issue for older adults there are limitations that must be considered. National surveillance data are useful to assess patterns related to health, however, the assessment of dietary intakes and quality is limited by the use of a singular 24–hour recall that may not reflect usual intakes. Also, the single day of recall for dietary intake may be subjective to recall bias and social desirability bias, with the potential for under– and over–reporting of food intakes. In addition, physical functioning limitations are self–reported. Furthermore, these analyses do not represent nutritional adequacy, but meeting recommended intake levels and cannot be interpreted as causal for functional outcomes. However, patterns of nutrient intakes for those meeting or not meeting protein recommendations utilizing a nationally representative sample illuminate broader nutritional and dietary pattern concerns in the aging population.

## Conclusion

Overall diet quality among adults aged 51 years and older needs improvement and dietary protein intakes are falling below the current recommendation level. Individuals not meeting the protein recommendation also have lower diet quality, and are more likely to not meet recommended intakes for micronutrients which have antioxidant or immune modulating activity including zinc, selenium, vitamin E, vitamin C, and vitamin D. Furthermore, not consuming enough protein limits functionality and may result in lower muscle mass. Future research is needed to assess the association between protein intakes, diet quality, and physical functioning longitudinally among the growing aging population.

The associations between reduced protein intakes, poorer diet quality, aging, and physical functioning reported here could help guide dietary counseling for those 51 years or older. Nutrition screening procedures should not be limited to the oldest adults and could begin with those over 50 years of age.

## ClinicalTrials.gov Identifier

NCT01275365 Financial Disclosures: Authors (CMA, MRS, SB, ACM, MS, SB, LLM) report grants from National Institutes of Health, nonfinancial support from Abbott Laboratories, nonfinancial support from Bariatrix Nutrition Corporation, and non-financial support from National Dairy Countil, to their institutions during the conduct of the study. Dr. Apovian reports personal fees from Nutrisystem, personal fees from Zafgen, personal fees from Sanofi-Aventis, grants and personal fees from Orexigen, personal fees from NovoNordisk, grants from Aspire Bariatrics, grants and personal fees from GI Dynamics, grants from Myos, grants and personal fees from Takeda, personal fees from Scientific Intake, grants and personal fees from Gelesis, other from Science-Smart LLC, personal fees from Merck, personal fees from Johnson and Johnson, grants from Vela Foundation, grants from Dr. Robert C. and Veronica Atkins Foundation, grants from Coherence Lab, grants from Energesis, grants from PCORI, outside the submitted work. Dr. Bhasin reports grants from MIB, grants from AbbVie, grants from Transition Therapeutics, during the conduct of the study; personal fees from Novartis, personal fees from AbbVie, other from FPT, outside the submitted work. In addition, Dr. Bhasin has a patent on method of determining free testosterone pending. Dr. Campbell reports grants and personal fees from National Dairy Council, grants and non-financial support from National Cattlemen’s Beef Association, grants from American Egg Board, grants from National Pork Board, and personal fees from Coca-Cola Foundation, outside the submitted work.
